# Growth mechanism of carbon nanotubes: a nano Czochralski model

**DOI:** 10.1186/1556-276X-7-356

**Published:** 2012-07-01

**Authors:** Jingyu Lu, Jianmin Miao

**Affiliations:** 1 School of Mechanical and Aerospace Engineering, Nanyang Technological University, Singapore, 639798, Singapore

**Keywords:** Carbon nanotube, Growth mechanism, Czochralski, Nano-CZ, catalytic, Nanowire

## Abstract

Carbon nanotubes (CNTs) have been under intense investigations during the past two decades due to their unique physical and chemical properties; however, there is still no commonly accepted growth mechanism to describe the growth behavior of CNTs. Here, we propose a nano Czochralski (CZ) model which regards the catalytic growth of a CNT as a CZ process taking place on the nano scale. The main idea is that, during the CNT growth, each catalyst particle acts as a nano crucible to nucleate and maintain the CNT growth, and the extruding CNT rotates relative to the nano crucible, leading to a chirality-dependent growth rate. In this case, the structural quality gradually changes along the CNT due to the dynamic generation-reconstruction-diffusion of defects during the CNT growth. The nano CZ mechanism may also apply to the catalytic growth of many other one-dimensional (1D) nanostructures (including various nanotubes and nanowires), thus further efforts will be stimulated in the quality and property control, as well as application explorations of these 1D nanomaterials.

## Background

The *sp*^2^ hybridized carbon atoms organized in one-dimensional (1D) tubular structure offer carbon nanotubes (CNTs) astonishing physical and chemical properties, which have been motivating numerous research efforts from both academic and industrial communities for more than two decades. However, the fundamental growth mechanism is still a matter of on-going research [1], though it is of great significance in the understanding and control of CNT quality, properties, and applications. The prevailing vapor-liquid-solid (VLS) mechanism was first proposed by Wagner and Ellis for the growth of silicon whiskers from gold droplets [2], and it is widely used to explain the growth of CNTs [3] and other 1D nanomaterials [4], but it is still controversial and under development [5]. Another model attracting growing attention is the vapor-solid-solid (VSS) mechanism proposed by Persson et al. to explain the growth of GaAs nanowires [6], and later, the VSS mechanism was applied to the growth of CNTs as well [7]. The main difference between the VLS and VSS mechanism lies in the phase (either liquid or solid) of the catalyst particle during the CNT growth; however, both models neglect some other effects of the growth (e.g., the catalyst rotation and the chirality-dependent growth). Here, we propose a nano Czochralski (CZ) growth mechanism and try to fill this gap.

## Presentation of the hypothesis

**Figure 1:**
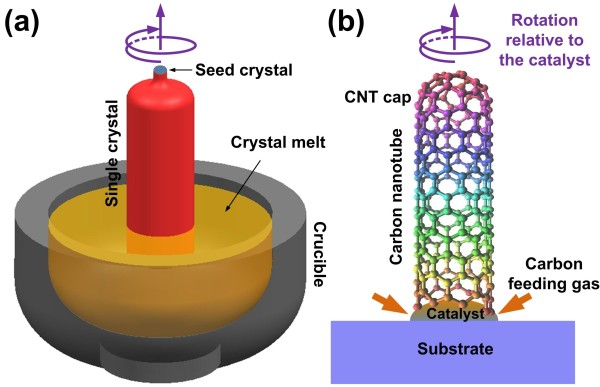
** Schematic of a conventional Czochralski growth process and the nano-CZ model.** Schematic of (**a**) conventional Czochralski growth process of a macroscopic single crystal, and (**b**) the nanoCZ model for the base-type growth of a CNT.

The CZ process is named after Jan Czochralski who discovered it in 1916, it enables the mass production of high quality single crystal materials (especially silicon) with the ingot diameter up to 300 mm [8], which lays the foundation for the prosperity of semiconductor industry and modern electronics. The basic idea of the CZ process is to pull a seed crystal rotationally from the melt of the single crystal in a crucible as illustrated in Figure 1a.

In a similar way, if we regard the CNT growth as a CZ process taking place on the nanoscale, then the role of the crystal seed in CZ process will be played by the CNT cap, while each catalyst particle will act as a nano crucible, and the corresponding nano-CZ mechanism can be sketched as shown in Figure 1b. In the beginning, the nano crucible absorbs and reacts with the environmental carbon feeding gas (usually hydrocarbon gas, like C_2_H_2_[9]) at high temperatures, so as to generate highly mobile carbon atoms (instead of the carbon melt, like the silicon melt in CZ process) continuously until it is oversaturated, when a CNT cap starts to nucleate. The formation of the hemispherical CNT cap from the catalyst reduces the surface energy of the system because the basal plane of the graphite is much more stable than those dangling bonds [10]; in a similar way, the cap tends to flatten to minimize the system energy, which finally leads to the lift-off of the cap from the catalyst and starts the extrusion of CNTs [11]. In the CZ process, the crystal seed is provided before the start of the crystal growth; while in the nano-CZ model, the ’seed’ cannot be found until the formation of the CNT cap. The nano crucible functions like a transitional chemical reservoir of highly mobile carbon atoms between the environmental feeding gas and the extruding CNT, while the role of pulling force in CZ process is played by the extrusion force from the continuous CNT precipitation in the nano-CZ model, which is estimated to be 0.16 nN per CNT shell [12]. Similarly, the CNT rotates relative to the catalyst particle during the CNT extrusion, which has been observed *in situ* with the field emission microscopy by Marchand et al. [13]. In this regard, the growth of a chiral CNT can be regarded as a screw extrusion process (as predicted by Ding et al. [14] in 2009) relative to its catalyst instead of a linear elongation as generally assumed, and carbon atoms are precipitated and added to the CNT shell circumferentially atom by atom [13]. During the screw extrusion process, the precipitation rate of carbon atoms and the rotation velocity of the CNT relative to the catalyst will determine the helix angle and thus the chiral index of the CNT shell, thus the CNT growth rate is chirality-dependent, which was predicted by Dumlich et al. theoretically [15] and was recently confirmed experimentally by Rao et al. [16]. It has been suggested that chirality-selected CNTs can be obtained through proper control of catalyst particles [17-19]; for example, Chiang and Sankaran have shown that the catalyst composition can be used to adjust the chirality distribution of CNTs [20]. In addition, since the CNT extrusion in the nano-CZ model is driven by the precipitation of the CNT, to grow CNTs of the desired properties besides the traditional method of adjusting growth operational parameters, it is promising to introduce external forces (e.g., through electrical or magnetic field) to guide the catalyst behavior and control the CNT growth process. Recent studies have shown that the introduction of an electric to the CNT growth could lead to CNTs with higher metallic conduction behavior [21], and the magnetic field can be applied to improve the CNT structural quality during the CNT growth process [22]. On the other hand, the product of the conventional macroscale CZ process is a solid single crystal cylinder, while in the nano-CZ growth of CNTs, here, the outcome is a hollow tubular structure, because it is energetically favored during the precipitation of carbon on the nanoscale [23]. Note that although Figure 1b only illustrates the root-type growth mode with the catalyst particle staying at the CNT root during the CNT growth, the basic idea of nano CZ applies to the tip-type growth as well, only that the catalyst particle stays at the tip of a CNT instead.

In the nano-CZ process, the role of crucible can be played by both metal and non-metal nanoparticles; and the nano crucible can be in solid or liquid state, on the condition that it can continuously provide highly mobile carbon atoms from the environment and nucleate the CNT precipitation. So far, various metal [24-26] and non-metal [27,28] nanoparticles have been found capable of catalyzing the CNT growth. Therefore, in a sense, the VLS and VSS mechanism may be regarded as two special cases of the nano-CZ mechanism, and there may be some other cases beyond the reach of VLS and VSS mechanism but within the scope of the nano-CZ mechanism. However, the nano-CZ mechanism may have some limitations as well; the detailed dynamics of the nucleation and screw extrusion process of a CNT require more in-depth investigations, which may lead to an exciting landscape ahead.

## Testing the hypothesis

If the proposed nano-CZ mechanism is applicable, we would expect a longitudinally changed structural quality (or defect concentration) distribution along the as-grown CNT (instead of uniform quality as generally assumed). The longitudinal uneven quality distribution is common in macro single crystal silicon obtained via the conventional CZ process [29,30], which was explained by Voronkov in 1982 [31]; briefly speaking, his model predicts the distribution of point defects (interstitials or vacancies) in CZ-grown single crystals by considering the defects generation, recombination, and transportation, and it assumes a constant defects concentration at the precipitation interface and immediate recombination of interstitials with vacancies. However, in nano-CZ growth of CNTs here, the defect system seems to be more complicated, it involves not only the annihilations of adatoms with vacancies but also their reconstructions into other defects; in addition, the catalyst activity decays as the growth proceeds instead of a constant crucible in CZ process. In this case, we would anticipate that the CNT defects experience a dynamic generation-reconstruction-diffusion (GRD) process during the catalytic growth of CNTs.

During the nano-CZ process, there are many factors that may introduce structural defects to CNTs. First, the anisotropic nature of the catalyst catalytic activity often results in an uneven CNT spatial extrusion hodograph [32], thus carbon atoms are not added to the CNT shells circumferentially with the same speed, which could lead to the formation and accumulation of stress in CNTs, together with the morphological reconstruction (e.g., elongation) of the catalyst particle during the growth [33], defects will be generated. Second, the CNT extrusion rate and relative rotation speed should maintain certain relations to keep the chiral vector of each CNT shell constant, or it will induce further defects and may lead to the longitudinal chirality evolution, which invite more in-depth researches. Third, the catalyst activity decreases as the CNT growth proceeds [34], thus the CNT precipitation rate gradually decreases, while the carbon supply is almost constant from the environment; when the gas adsorption rate surpasses the CNT precipitation rate, extra carbon atoms will precipitate in the form of defects at the CNT-catalyst interface [35]; some defects stay on the catalyst particle surface and decrease its activity further, thus more and more defects will come out to cover the catalyst particle, which accelerates the termination of the CNT growth and leads to an increasingly higher defects concentration at the newly-grown CNT segment. Therefore, defects can hardly be eliminated during the catalytic growth of CNTs, especially those long CNTs.

Some defects can be reconstructed or even repaired thermodynamically during the growth [36]. It is generally accepted that the catalyst particle can heal defects with the consumption of adatoms or amorphous carbon [37]; the recent high temperature *in situ* observations by Asaka et al. demonstrate the graphitization of amorphous carbon into CNTs without catalyst [38], indicating that the high temperatures also contribute to the repair of CNT structures. In addition, similar to the conventional CZ process, vacancies can annihilate with adatoms to generate pristine CNT structures [39], or alternatively, they propagate and aggregate into clusters and lead to discontinuities in CNT shells [40], and some of them may even reconstruct into the pentagon-heptagon defect [41].

These effects will lead to a defect concentration gap between the CNT precipitation interface and the other CNT end, which drives the diffusion of defects along the CNT. For example, Li et al. [42] found that the carbon adatoms could move easily along the graphene lattice with the diffusion barrier of approximately 0.76 eV, thus adatoms can propagate and recombine with vacancies by overcoming the activation energy of 0.89 eV even at room temperature, let alone at higher temperatures during the CNT growth. Therefore, the axial defects diffusion will lead to a concentration gradient along the CNT. Upon the birth of defects, their generation, reconstruction, and diffusion may take place simultaneously during the CNT growth process, though their respective rates may change as the growth proceeds. The longitudinally changed CNT quality has been confirmed experimentally in recent studies [43-45].

## Implications of the hypothesis

In summary, we proposed the nano-CZ growth mechanism for the catalytic growth of CNTs. Within the nano-CZ mechanism, the catalyst particle acts as a nano crucible to nucleate and maintain the CNT growth, a chiral CNT grows in a screw extrusion manner. The nano-CZ model will lead to an uneven quality distribution along the CNT as confirmed by many groups [43-45], and we attribute the phenomenon mainly to the dynamic GRD of defects in the CNT during its growth. It should be noted that there may be certain limitations to the nano-CZ mechanism, and the details of the CNT screw extrusion and the defect dynamics need further in-depth investigations. The proposed nano-CZ mechanism may also apply to the catalytic growth of many other 1D nanostructures; the recent growths of ZnO nanotubes and nanowires have shown similar behaviors [46]. Since material properties strongly depend on the corresponding structural quality, local physical and chemical properties are expected to change along these 1D nanostructures. By exploring the nano-CZ model further, it is promising to obtain CNTs and many other 1D nanomaterials with desired properties for various applications.

## Abbreviations

CNT, carbon nanotube; CZ, Czochralski; 1D, one dimensional; GRD, generation-reconstruction-diffusion; VLS, vapor-liquid-solid; VSS, vapor-solid-solid.

## Competing interests

The authors declare that they have no competing interests.

## Authors’ contributions

JL drafted the article and did the literature survey and analysis; JM participated in the design and critical revision of the manuscript. All authors read and approved the final manuscript.

## Authors’ information

JL is a PhD candidate under Professor JM in the School of Mechanical and Aerospace Engineering, Nanyang Technological University, Singapore.
